# Distribution of energy in the ideal gas that lacks equipartition

**DOI:** 10.1038/s41598-023-30636-6

**Published:** 2023-02-28

**Authors:** Dmitry M. Naplekov, Vladimir V. Yanovsky

**Affiliations:** 1 Institute for Single Crystals, NAS Ukraine, 60 Nauky Ave., Kharkov, 61001 Ukraine; 2 V. N. Karazin Kharkiv National University, 4 Svobody Sq., Kharkov, 61022 Ukraine

**Keywords:** Statistics, Statistical physics

## Abstract

The energy and velocity distributions of ideal gas particles were first obtained by Boltzmann and Maxwell in the second half of the nineteenth century. In the case of a finite number of particles, the particle energy distribution was obtained by Boltzmann in 1868. However, it appears that this distribution is not valid for all vessels. A round vessel is a special case due to the additional integral of motion, the conservation of the gas angular momentum. This paper is intended to fill this gap, it provides the exact distribution of particle energy for a classical non-rotating ideal gas of a finite number of colliding particles in a round vessel. This previously unknown distribution was obtained analytically from the first principles, it includes the dependence on all the particle masses. The exact mean energies of gas particles are also found to depend on the system parameters, i.e., the distribution of energy over the degrees of freedom is not uniform. Therefore, the usual ideal gas model allows for the uneven energy partitioning, which we study here both theoretically and in simple numerical experiments.

An ideal gas of non-interacting particles is a classical model system to study the statistical behavior of systems of many particles. A number of classical results^1–4^ are related to this case, such as the Boltzmann or the Gibbs distributions $p(E) \sim e^{-\frac{E}{k T}}$, the Maxwell distribution of particle velocities, etc. A theorem about the uniform distribution of energy over the degrees of freedom^5–7^ was proved for many, but not all systems. The exact limits of its applicability are still being debated^8,9^. There are also new interesting results, such as the Jarzynski relations^10^, fluctuation theorems^11^, etc.

Usually, the ideal gas is considered in a thermodynamic limit and the number of its degrees of freedom is infinite. The total energy is infinitely large and there is a non-zero probability for a particle or some subsystem to have any arbitrarily large energy. An essentially different is the more general case of an ideal gas consisting of a finite number of particles with a finite number of degrees of freedom. The entire energy of such a gas in an isolated vessel is finite. A particle or a subsystem cannot have its energy higher than the total system energy. Therefore, the particle velocity and energy distributions proceed only to some finite values, and then they are exactly zero. In particular, for a two-dimensional ideal gas of a finite number of particles, Boltzmann in his classical paper^2^ obtained the particle energy distribution $p_{\text {Bol}}^{\{N\}}(E)=(N-1)\frac{(E_{\text {tot}}-E)^{N-2}}{E_{\text {tot}}^{N-1}}$ at $E \le E_{\text {tot}}$ and $p_{\text {Bol}}^{\{N\}}(E)=0$ at $E>E_{\text {tot}}$, where *N* is the number of particles, and $E_{\text {tot}}$ is their total energy. This distribution does not depend on the masses of particles and corresponds to equal mean particle energies.

Systems with a finite number of degrees of freedom are well related to very small objects, like many-atomic molecules, which are actively studied currently. It may be biological machines, such as monomolecular motors^12,13^, whose operating principles are currently not fully understood. These individual molecules are able to efficiently produce mechanical work, acting at an energy level of the order of thermal energy. The transport properties of molecules are being actively studied^14,15^. For example, the stochastic motion of $C_{60}$ molecules over the surface of graphene in^14^.

In many of such^16–23^ and other^24,25^ systems, deviations from the energy equipartition have been observed. In molecular dynamics simulations of the behaviour of biomolecules, such as proteins or peptides, the mean energies of solute macromolecules are often found to be lower than those of the solvent molecules. This deviation from energy equipartition has received a special name: the “Hot-Solvent/Cold-Solute” problem^26,27^. One of the reasons for it is connected with the conservation of the total system momentum^28^. Absence of the energy equipartition is reported for insulated clusters of atoms^17,18^, massive particles immersed into a molecular gas^19^. It is of high interest in systems of confined, laser-cooled atoms^20–22^, etc. For the better understanding of such behaviour, simple model cases are important, which demonstrate the ground physical effects. Such a new case is considered in this paper, showing theoretically how exactly the energy is partitioned in the ideal gas that lacks equipartition.

## Gas in a round vessel

We will consider the two-dimensional motion of a finite number of colliding particles *N*, placed in a stationary circular vessel of radius *R*. All particles will be of a round shape with radii $r_i$ and masses $m_i$, generally different. The motion of particles will be rectilinear and uniform. All collisions between particles and with the vessel walls will be absolutely elastic. All particle masses and energies will be normalized to the total gas mass $M_{\text {tot}}$ and energy $E_{\text {tot}}$.

**Figure 1:**
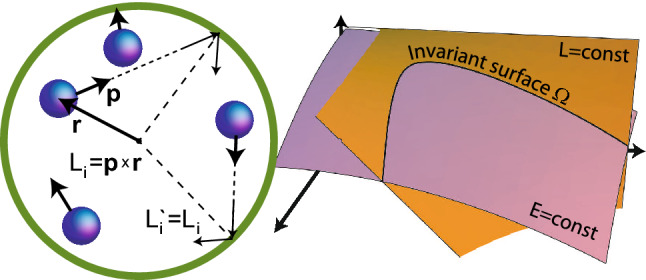
A stationary round vessel of radius *R* contains *N* colliding particles with masses $m_i$ and radii $r_i$. The reflection of a particle from the vessel walls does not change its angular momentum $L'_i=L_i$, due to the boundary symmetry. As a result, the total gas angular momentum is conserved, unlike the other vessels. The surface of constant energy is not filled densely. The system trajectory instead fills the invariant surface $\Omega$. For this reason, the behaviour of the ideal gas in the round vessel is very different.

In collisions with the walls of a stationary vessel, the energy of the particle after reflection is equal to its energy before the collision, but the momentum of the particle changes upon reflection. The angular momentum of a particle also generally changes, but the round vessel is a special case. After reflection from any point of its boundary, the particle’s angular momentum $L'_i$ remains equal to its initial angular momentum $L_i$ (see Fig. 1). In particle collisions, it is also conserved. As a result, the value $L_{\text {tot}}=\sum _{i=1}^{N} \left( y_i p_{x i} - x_i p_{y i}\right)$ remains constant during evolution. This distinguishes a round vessel (axisymmetric vessels in the 3D case) from all other possible vessels. Further, we will consider a non-rotating ideal gas with $L_{\text {tot}}=0$.

During evolution, the point representing the state of an isolated system in a phase space stays on the surface, which is called invariant. For an ideal gas in a round vessel, this surface is the intersection of surfaces of constant energy and constant angular momentum. It makes the system non-ergodic in a sense that the surface of constant energy is not filled densely. The only essential assumption in our theoretical consideration is that instead the invariant surface $\Omega$ is densely filled.

**Figure 2:**
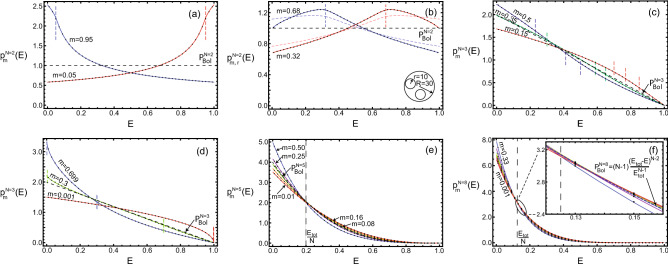
Energy distributions for $N=(2,3,5,8)$ ideal gas particles with masses *m* in a round vessel. They are obviously different for different mass particles, contrary to the Boltzmann distribution. The continuous curves show theoretical distributions according to (**a**) Eq. (5), (**c**, **d**) Eq. (6) and (**e**, **f**) Eq. (3), the dots show the results of the numerical experiments. The vertical dotted lines indicate the distribution segments boundaries. The radii of all particles were small compared to the vessel size ($r_i=2$ and $R=40$), except for the case (**b**) where $r_i=10$ and $R=30$. For big enough particles, their size affects the energy distribution, the theoretical distributions with and without (Eq. (5) by dotted line) the particle size amendment are shown. As the number of particles increases, the typical energy distribution approaches the Boltzmann one, shown by the dotted lines. The deviations from it correlate with the particle mass, the distributions of light particles pass below the Boltzmann one at $E \lesssim \frac{E_{\text {tot}}}{N}$ and above it otherwise.

## Particle energy distributions

Let us consider the energy distribution of ideal gas particles in the round vessel. For the derivation of its analytical form, we have first calculated the density of filling of an invariant surface $\Omega$. The filling density of an invariant surface of a Hamilton system is known to be uniform with respect to the special measure, which is called ergodic. For the surface of constant energy, it defines the hypervolume of an elementary surface part as $d \Omega = \frac{\textit{const} \; d \Sigma }{|grad E|}$. Similarly, for the intersection of the surfaces of constant energy and constant angular momentum, we obtain the following filling density:

1 $$\begin{aligned} d p_{\Omega } = \frac{\textit{const} \; \; d \Sigma }{\sqrt{|grad E|^2 \; |grad L|^2 - \left( grad E \cdot grad L\right) ^2}} \end{aligned}$$

This is the probability density per elementary hypervolume $d \Sigma$ (usual Euclidean measure) of a curvilinear $4 N-2$-dimensional hypersurface. It was calculated with the use of the Liouville theorem, which comes from the laws of particle motion. After the procedure of “projection” of this probability density, to make the integration over the phase coordinated possible, and substitution of the explicit expressions for energy and angular momentum, we came to the following probability density (at $L_{\text {tot}}=0$):

2 $$\begin{aligned} d p = \frac{\textit{const} \quad dx_1 .. dp_{y N-1}}{\sqrt{m_N \left( x_N^2+y_N^2\right) \left( 2 E_{\text {tot}}-\underset{i=1}{{\mathop {\sum }\limits ^{N-1}}} \frac{p_{x i}^2+p_{y i}^2}{m_i}\right) - \left( \underset{i=1}{{\mathop {\sum }\limits ^{N-1}}} \left( p_{y i} x_i - p_{x i} y_i\right) \right) ^2} } \end{aligned}$$

for the coordinates of gas particles to be $x_1..x_N$, $y_1,..,y_N$ and the particle momenta components to be $p_{x 1}..p_{x N-1}$, $p_{y 1 }..p_{y N-1}$. The last two components are determined by the laws of conservation. Integration of this expression over the phase variables within appropriate limits brings the desired gas distributions. All of the integration limits are finite, and they account for the fact that a particle with mass $m_1$ and energy *E* cannot be found at any point inside the vessel. It can only be located inside the strip of width $2 \sqrt{\frac{\left( E_{\text {tot}}-E\right) \left( \sum _{i=2}^{N} m_i \left( x_i^2+y_i^2\right) \right) }{m_1 E}}$, oriented in the direction of the particle’s momentum. Otherwise, the remaining energy will not be enough for other particles to compensate for the angular momentum of this particle. Finally, for the energy distribution of a particle of mass $m_1$, we obtained:

3 $$\begin{aligned} P_{m_1}^{\{N\}}(E) = \underset{\Lambda }{\int \int }\ A \frac{ \left( \left( E_{\text {tot}}-E\right) J\left( R_2,..,R_N\right) -E m_1 y_1^{*2}\right) ^{N-\frac{5}{2}} }{J\left( R_2,..,R_N\right) ^{N-2}} \sqrt{R^2-y_1^{*2}} \underset{n=2}{{\mathop {\prod }\limits ^{N}}}R_n \quad d y_1^* d R_2..d R_N \end{aligned}$$

Here $J\left( R_2,..,R_N\right) =\underset{n=2}{{\mathop {\sum }\limits ^{N}}} m_n R_n^2$ is the moment of inertia of other particles, *A* is the normalization constant. This is the most compact representation of the exact general energy distribution that we have found. In Eq. (3), the integration over momenta components is already done, while the integration over coordinates remains. The area of this integration $\Lambda$ is:

4 $$\begin{aligned} \Lambda = \{ (y_1^{*},R_2,..,R_N) : |y_1^{*}|<\sqrt{\frac{E_{\text {tot}}-E}{E m_1} J\left( R_2,..,R_N\right) }, \quad y_1^{*} \in (-R, R), R_n \in (0, R), n \in [2, N] \} \end{aligned}$$

This area is the intersection of the interior of the rectangular region $y_1^{*} \in (-R, R), R_n \in (0, R), n \in [2, N]$ and the interior of the hypersurface $\underset{n=2}{{\mathop {\sum }\limits ^{N}}} m_n R_n^2 - \frac{E m_1}{E_{\text {tot}}-E} y_1^{*2}=0$. Depending on the range of energies *E*, one of these areas can be wholly or partially inside the other, or wholly include the other. In each of these cases, corresponding to different particle energy regions, the limits of integration and the resulting distribution function are different. As a result, the particle energy distribution turns out to consist of some finite number of segments, each with its own function that can be calculated explicitly.

In the case of two particles, their energy distribution is the most simple. It consists of two sites, and for the particle $m_1$ has the following form:

5 $$\begin{aligned} P^{\{\text {N=2}\}}_{m_1}(E)=A {\left\{ \begin{array}{ll} \frac{\left( \left( m_1 - m_2\right) E + m_2 E_{\text {tot}}\right) }{E \sqrt{m_1 \left( E_{\text {tot}}-E\right) }}E \left( \sqrt{\frac{m_1 E}{m_2 \left( E_{\text {tot}}-E\right) }}\right) +\frac{\left( \left( m_1 + m_2\right) E - E_{\text {tot}} m_2\right) }{E \sqrt{m_1 \left( E_{\text {tot}}-E\right) }}K\left( \sqrt{\frac{m_1 E}{m_2 (E_{\text {tot}}-E)}}\right) \\ \qquad \qquad \qquad \qquad \qquad \qquad \qquad \qquad \qquad \qquad \qquad \qquad \qquad \qquad E \in \left( 0,\frac{m_2}{m_1+m_2} E_{\text {tot}}\right) \\ \\ \frac{\left( (m_1 - m_2) E + m_2 E_{\text {tot}}\right) }{(E_{\text {tot}}-E) \sqrt{m_2 E}}E\left( \sqrt{\frac{m_2 (E_{\text {tot}}-E)}{m_1 E}}\right) -\frac{\left( (m_1 + m_2) E - E_{\text {tot}} m_2 \right) }{\left( E_{\text {tot}}-E\right) \sqrt{m_2 E}}K\left( \sqrt{\frac{m_2 (E_{\text {tot}}-E)}{m_1 E}}\right) \\ \qquad \qquad \qquad \qquad \qquad \qquad \qquad \qquad \qquad \qquad \qquad \qquad \qquad \qquad E \in \left( \frac{m_2}{m_1+m_2} E_{\text {tot}}, E_{\text {tot}}\right) \end{array}\right. } \end{aligned}$$

where $E(k)=\int _{0}^{\pi /2} \sqrt{1+k^2 \sin ^2 \Theta } d\Theta$ and $K(k)=\int _{0}^{\pi /2} \frac{d\Theta }{\sqrt{1+k^2 \sin ^2 \Theta }}$ are complete elliptic integrals. This distribution is symmetrical $P^{\{{\text {N=2}}\}}_{m_2}(E)=P^{\{{\text {N=2}}\}}_{m_1}(E_{\text {tot}}-E)$, since if the first particle has energy *E*, then the energy of the second particle will be $E_{\text {tot}}-E$, the corresponding probabilities are equal.

Some examples of the energy distributions for two particles are shown in Fig. 2a, b. The continuous lines show the theoretical distributions, the dots show the distributions obtained via numerical simulation of particle motion. The sites of the distribution Eq. (5) are smoothly glued together, but the way distribution goes changes with the transfer from one site to another. The distributions for two particles clearly demonstrate that the theoretical subdivision of the energy distribution on segments corresponds to the physical constitution of these distributions, i.e., it is not just a mathematical description feature. The physical reason for the change in the distribution behaviour is that, starting from a certain energy, restrictions appear on the possible combinations of motion and location of the particle.

The distribution Eq. (3) was derived for ideal gas particles, i.e., particles of negligible size. However, if the particles are large enough compared to the vessel size, for example $r_i=10$ and $R=30$ as in Fig. 2b, then the distribution obtained experimentally will significantly differ from Eq. (5). In other words, the particle energy distribution in a round vessel depends not only on the masses but also on the sizes of particles. An amendment, accounting for the particle sizes, can also be calculated. It is necessary to integrate the integrand from Eq. (3) over the area where the particles intersect with each other or with the vessel walls. The resulting amendment must be subtracted from the original distribution before it is normalized.

In the case of three particles of negligible sizes, the energy distribution of a particle $m_1$ at $m_2>m_3$ can be obtained explicitly as:

6 $$\begin{aligned} P^{\{{\text {N=3}}\}}_{m_1}(E)=A {\left\{ \begin{array}{ll} p(m_2 + m_3) - p(m_2) - p(m_3) \quad E \in \left( 0,\frac{m_3 E_{\text {tot}}}{m_1+m_3}\right) \\ p(m_2 + m_3) - p(m_2) - p^*(m_3) \quad E \in \left( \frac{m_3 E_{\text {tot}}}{m_1+m_3} , \frac{m_2 E_{\text {tot}}}{m_1+m_2} \right) \\ p(m_2 + m_3) - p^*(m_2) - p^*(m_3) \quad E \in \left( \frac{m_2 E_{\text {tot}}}{m_1+m_2} , \frac{(m_2+m_3) E_{\text {tot}}}{m_1+m_2+m_3} \right) \\ p^*(m_2 + m_3) - p^*(m_2) - p^*(m_3) \quad E \in \left( \frac{(m_2+m_3) E_{\text {tot}}}{m_1+m_2+m_3} , E_{\text {tot}}\right) \\ \end{array}\right. } \end{aligned}$$

where the functions *p*(*m*) and $p^*(m)$ are:

7 $$\begin{aligned}{} & {} p(m)=\frac{\sqrt{m}}{E \sqrt{m_1(E_{\text {tot}}-E)}} \Bigg ( \left( (E_{\text {tot}} - E)^2 m^2 + 14 E m_1 (E_{\text {tot}} - E) m + E^2 m_1^2\right) E\left( \sqrt{\frac{m_1 E}{m (E_{\text {tot}}-E)}}\right) \nonumber \\{} & {} \quad -(E_{\text {tot}} m - E (m_1 + m)) ((E_{\text {tot}} - E) m + 7 E m_1) K\left( \sqrt{\frac{m_1 E}{m (E_{\text {tot}}-E)}}\right) \, \Bigg ) \nonumber \\{} & {} p^*(m)=\frac{1}{(E_{\text {tot}}-E) \sqrt{E}} \Bigg ( \left( (E_{\text {tot}} - E)^2 m^2 + 9 E m_1 (E_{\text {tot}} - E) m - 4 E^2 m_1^2\right) E\left( \sqrt{\frac{m (E_{\text {tot}}-E)}{m_1 E}}\right) \nonumber \\{} & {} \quad +(E_{\text {tot}} m - E (m_1 + m)) (7 (E_{\text {tot}} - E) m - 4 E m_1) K\left( \sqrt{\frac{m (E_{\text {tot}}-E)}{m_1 E}}\right) \, \Bigg ) +\nonumber \\{} & {} \quad +\frac{15 \pi m}{64 \sqrt{E}} \left( 16 E m_1 -(E_{\text {tot}} - E) m \left( _4F_3\left( {\textbf {a}}_1;{\textbf {b}};\frac{m (E_{\text {tot}}-E)}{m_1 E}\right) +_4F_3\left( {\textbf {a}}_2;{\textbf {b}};\frac{m (E_{\text {tot}}-E)}{m_1 E}\right) \right) \right) \end{aligned}$$

Here $_pF_q({\textbf {a}};{\textbf {b}};z)$ is a generalized hypergeometric function defined as $_pF_q({\textbf {a}};{\textbf {b}};z)=\underset{k=0}{{\mathop {\sum }\limits ^{\infty }}} \frac{(a_1)_k ... (a_p)_k}{(b_1)_k ... (b_q)_k} \frac{z^k}{k!}$, $(a)_n=a(a+1)..(a+n-1)$. Its parameters are: ${\textbf {a}}_1=(1/2, 1, 1, 3/2)$, ${\textbf {a}}_2=(1, 1, 3/2, 3/2)$, ${\textbf {b}}=(2, 2, 3)$.

Typical distributions for three particles are shown in Fig. 2c, d. Each curve corresponds to the choice of one of the particles, the mass of which was substituted into Eq. (6) as $m_1$, the heaviest of the remaining particles as $m_2$, and the last one as $m_3$. For three or more particles, the subdivision of their distributions on segments is no longer obvious from the distribution appearance. But its reasons are the same as in the case of two particles.

Examples of distributions for more particles, obtained by numerical integration of Eq. (3) are shown in Fig. 2e, f. It is clear that with an increase in the number of particles, the distributions of particles with different masses approach each other and the corresponding Boltzmann distribution. The vertical dotted line at Fig. 2e, f shows the characteristic energy value $\frac{E_{\text {tot}}}{N}$. The probability of having this energy is approximately equal for all particles. The energy below it is more often for heavy particles, while light particles are more likely to have energies above $\frac{E_{\text {tot}}}{N}$. As it turns out, the smaller the particle mass, the higher its average energy (at $L_{\text {tot}}=0$).

In the case of a large number of particles of comparable masses, the moment of inertia of a single particle can be neglected compared to the moment of inertia of other particles. The energy of this particle *E* can also be considered small compared to the energy of all other particles $E_{\text {tot}}-E$. Under these assumptions, we may consider that $\frac{(E_{\text {tot}}-E) J(R_2,..,R_N)-E m_1 y_1^{*2}}{J(R_2,..,R_N)} \approx (E_{\text {tot}}-E)$ and $J(R_2,..,R_{N}) \approx J(R_2,..,R_{N-1})$. Then from the distribution Eq. (3) one can obtain that $P_{m}^{\{N\}}(E) \sim (E_{\text {tot}}-E) P_{m}^{\{N-1\}}(E)$. As a result, the limit of the distribution Eq. (3) coincides with the limit of the Boltzmann distribution $P_{\text {Bol}} \sim (E_{\text {tot}}-E)^{N-2}$ at $N \rightarrow \infty$. However, this is valid only if the particle mass $m_1$ allows for the term $E m_1 y_1^{*2}$ to be neglected. The distribution of heavy enough particle will still be different.

It is interesting to note that passage to the limits $N \rightarrow \infty$ and $E_{\text {tot}} \rightarrow \infty$ in the classical Boltzmann distribution $p_{\text {Bol}}^{\{N\}}(E)=(N-1)\frac{(E_{\text {tot}}-E)^{N-2}}{E_{\text {tot}}^{N-1}}$ yields the Boltzmann distribution $p_{\text {Bol}}(E)= \beta e^{-\beta E}$ for an infinite number of particles. Only here the temperature appears as the limit of the ratio $lim \frac{E_{\text {tot}}}{N} = \frac{1}{\beta } = k T$.

## Distribution of collision angles

Let us consider in more detail the stationary state of gas in a round vessel. At each collision of two particles, some energy is transferred from one particle to another. After equilibrium is established, the amount of energy received in collisions must, on average, be equal to the amount lost for every particle. Otherwise, the average energy of the particle will change in time. In a rectangular vessel, such a balance is achieved with the energy distributions of all particles being the same and, accordingly, with equal mean particle energies.

**Figure 3:**
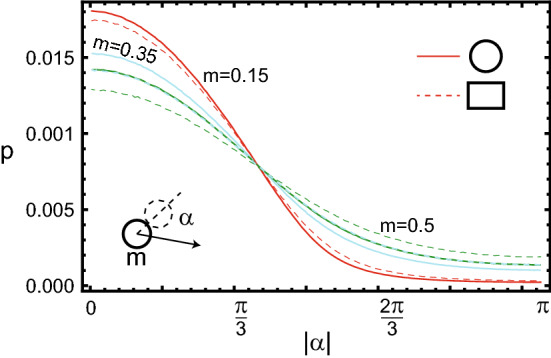
Distributions of collision angles for three particles with masses (*m* : 0.15, 0.35, 0.5) in a round vessel. For comparison, dotted lines show similar dependencies for a rectangular vessel. It is visible that in a round vessel, the share of frontal collisions with small $| \alpha |$ is higher. The distributions for $m_2=0.35$ in rectangular and $m_3=0.5$ in round vessels coincide.

Now we note that if any of the particles in the round vessel has some angular momentum *L*, then the remaining particles, in the case of a non-rotating gas, must have a total angular momentum $-L$. In other words, each of the particles moves against some counter flow since the gas of the remaining particles rotates in the opposite direction with respect to the selected particle. This leads to an increase in the share of frontal collisions for all particles. We consider numerically the distribution of particle collision angles, i.e., angles between the direction of motion of the selected particle and the direction to the centre of the particle with which the collision occurred. These distributions are shown in Fig. 3. For comparison, dotted lines show similar distributions for particles in a rectangular vessel. In round and rectangular vessels, these distributions are qualitatively the same, in contrast to the energy distributions. In a round vessel, particles collide frontally more often and catch up with one another less often than in a rectangular vessel. For light and fast-moving particles, for which frontal collisions initially predominate, the increase in their share is smaller than for heavier ones. However, the heavier the particle, and the fewer particles in the vessel, the greater this effect.

Thus, an additional law of conservation leads to a change in the distribution of particle collision angles. That leads to a change in the relationship between the energy transferred in collision and the average energies of particles. After equilibrium is reached, the average energy received and the average energy lost in collisions are set equal for every particle, not mean particle energies. Due to a different distribution of collision angles, in a round vessel, this balance is achieved with the mean particle energies being different.

## Average particle energy, equipartition violation

We now consider how the particle mean energy depends on the system parameters. Theoretically, this energy can be obtained using the $P_m^{\{N\}}(E)$ from Eq. (3) as:

8 $$\begin{aligned} <E>=\underset{E=0}{{\mathop {\int }\limits ^{E_{\text {tot}}}}} E P_m^{\{N\}}(E) dE \end{aligned}$$

This is an exact expression for particles of negligible size, it gives the mean particle energy as a definite integral. Unfortunately, even in the simplest cases, such integrals cannot be calculated analytically. Therefore, the exact analytical description of the energy partitioning is possible only in this general form, in quadratures.

In a round vessel, the average energy of a particle depends on all the parameters of the system. These parameters include the number of particles, all their masses, and all sizes. In addition, it also depends on the total angular momentum of the gas. Here, as above, we will consider a non-rotating gas $L_{\text {tot}}=0$. Among these parameters, several main ones can be distinguished, while the dependence on the rest, in most cases, is insignificant. The most important parameters (at $L_{\text {tot}}=0$) are the number of particles in the vessel *N* and the share of the selected particle mass $\frac{m}{M_{\text {tot}}}$ in the total mass of all particles $M_{\text {tot}}$.

**Figure 4:**
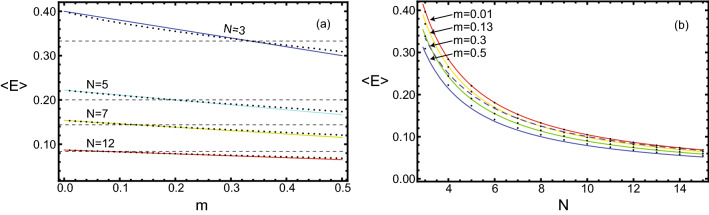
Dependence of the mean energy of selected particle: (**a**) on its mass *m* for a fixed number of particles in the vessel. (**b**) on the number of particles *N* for a fixed mass of selected particle. All other particles had equal masses. The dotted lines show the energy equipartition level. The dots show the average energy according to both Eq. (8) and the simulation of particle motion, the solid lines show the approximate formula Eq. (9). The dependence of the particle’s mean energy on its mass is very visible, in contrast to the energy equipartition.

Some dependencies of the particle mean energy on these parameters are shown in Fig. 4. These dependencies were constructed both by the numerical integration of Eq. (8) and by modelling the particles’ motion. The difference between the results obtained by these two methods was within the calculation errors. The Fig. 4a shows the dependencies of the particle average energy $<E>$ on its mass share *m* for a fixed number of particles $N=(3,5,7,12)$. It is visible that the smaller the mass of the particle, the greater its average energy. For light particles with masses less than the average $m<\frac{M_{\text {tot}}}{N}$, their average energies will be above the energy equipartition level, and vice versa for heavy particles. The Fig. 4b shows the change in particle average energy as the number of particles increases, provided that the mass share of the selected particle remains constant at $m=(0.01,0.13,0.3,0.5)$. As expected, as the number of particles increases, the energy of each decreases approximately in inverse proportion to *N*. The lower mass particles have higher energies at any number of particles in the vessel.

The calculated dependencies follow smooth curves and can be described by a general empirical formula of simple form. Approximately, the mean energy of an ideal gas particle of mass *m* in a round vessel with *N* particles (at $L_{\text {tot}}=0$) is given by the formula:

9 $$\begin{aligned} \frac{<E>}{E_{\text {tot}}} \approx \frac{2-\frac{m}{M_{\text {tot}}}}{2 N-1} \end{aligned}$$

This formula is obviously approximate, since it takes into account only the main parameters affecting the average energy. But if there are more than three particles and all of them have the same order masses and small sizes, then the formula Eq. (9) describes the observed distribution of energy between the particles well.

With an increase in the number of particles, their mass shares will become practically equal to zero if all particles are of comparable mass. As a result, their average energies become equal. However, if the particle’s mass share differs from zero, the average energy of such a massive, in a sense Brownian particle, will be lower than that of the surrounding particles, even with a large number of particles in the vessel.

## Summary

Usually, it is considered that the form of the vessel with ideal gas of colliding particles does not affect the gas behaviour. In this paper, we consider a special case when the circular vessel boundary leads to unusual, previously unknown distributions in the ideal gas of a finite number of particles. The mechanism of influence of the vessels’ shape is connected with the conservation of the gas angular momentum. Even in the absence of a circular gas flow, it changes the distribution of particle collision angles and all the other gas distributions.

We have obtained the exact analytical expressions for the distribution of energy of ideal gas particles in a round vessel. In a compact form, it is presented as the definite integral Eq. (3). This expression is valid at $L_{\text {tot}}=0$ for particles of negligible size but any number and mass. The explicit form of the energy distribution is complicated, we provide it for two and three particles for reference. As the number of particles increases, their energy distributions tend to the Boltzmann distribution. But the fewer particles in the vessel, and the greater the spread of their masses, the more significantly the energy distribution of ideal gas particles in a round vessel differs from the classical Boltzmann distribution.

The key difference of the round vessel is the uneven distribution of energy between gas particles. The exact theoretical expression for mean particle energy is the definite integral Eq. (8). There are two main parameters affecting the mean particle energy, the mass share of the particle and the number of particles in the vessel. The approximate dependence on these parameters is given by the simple formula Eq. (9). The greater the mass associated with the degree of freedom, the smaller its average energy will be. Even with a large number of particles, the mean energy of a sufficiently massive particle will be lower than that of the surrounding particles. These theoretical results have been strictly obtained and checked in numerical simulation experiments. They agree with numerous reports of equipartition violations in different systems. We expect them to be general for systems with a lack of energy equipartition.

## Data Availability

The details of the analytical or numerical calculations are available from the corresponding author on reasonable request.
